# Improved SH0 Guided Wave Transducers Based on Piezoelectric Fiber Patches †

**DOI:** 10.3390/s19132990

**Published:** 2019-07-06

**Authors:** Yongtak Kim, Tobias Gaul, Bernd Köhler

**Affiliations:** Fraunhofer IKTS, Maria Reich Str. 2, 01109 Dresden, Germany

**Keywords:** shear horizontal guided waves, structural health monitoring, SHPFP, piezoelectric fiber patch, directivity, mode purity

## Abstract

A piezoelectric fiber patch (PFP) is a transducer type that is suitable for guided-wave-based structural health monitoring (SHM) due to its light, thin, and flexible characteristics. In our previous work, a PFP-based transducer design for selective excitation of the zero-order shear horizontal wave mode (SH0) was introduced (shear horizontal PFP (SHPFP)). In this work, two modified SH0 wave PFP transducer designs are proposed: the rounded corner design and the dual design. The degree of improvement is determined by a numerical simulation and the dual design—the design with the most promise—is experimentally realized. Laser Vibrometry measured the generated wave field, confirming the results from the simulation. The new designs can generate an almost pure SH0 wave. The dual design has a very strong directivity that is useful for several guided-wave-based SHM applications. The conclusions on the design’s properties as a transmitter are also valid for its properties as a sensor due to the reciprocity of piezoelectric transducers.

## 1. Introduction

Guided elastic waves (GEWs) play an important role in both long-range ultrasonic testing and the structural health monitoring of plate- and shell-like structures. Objects can be tubes for fluids, such as oil or pipes for hot gases in power plants. Other interesting objects are foundation structures for offshore wind turbines or the skin of an aircraft’s fuselage. We can distinguish symmetric and antisymmetric Lamb waves and horizontally polarized shear waves in plate-like structures with isotropic material properties. For each type, there is an infinite series of modes that are characterized by their dispersion, which is the dependence of the phase velocity on the frequency.

Most GEW applications are based on Lamb waves, which have particle displacements in the sagittal plane. They are easily excited by piezoelectric wafer sensors and actuators (see [1] for an overview). Flexible interdigital polyvinylidene fluoride (PVDF) transducers have also been proposed [2]. These sensors have many advantages, such as a low weight and low price. However, it is difficult to excite a single mode with high purity. Additionally, the major challenge in the application of Lamb waves is their dispersive characteristics, which render complicated the use of localization methods based on the signal travel time. While there are methods that can be used to compensate for the dispersion [3], it is best to use wave modes, which are nondispersive. The fundamental order of the shear horizontal mode (SH0) fulfills this condition. Moreover, the SH0 is the only horizontally polarized plate mode for frequencies below the cutoff frequency of the next higher shear horizontal mode SH1. That is why efforts have been made to find efficient ways to generate and detect SH0 waves.

One way to generate shear waves is to apply surface traction or the corresponding volume force perpendicular to the intended propagation direction. On the receiving side, we have to detect the in-plane motion perpendicular to the propagation direction of the incoming wave. Several transducer types are available. Electromagnetic acoustic transducers (EMATs) are a well-known example [4,5]. They excite the wave directly in the object based on the conductivity and the magnetic properties of metals. One disadvantage of EMATs is that their application is restricted to metals. For structural health monitoring (SHM) applications, another disadvantage is their rather large size and heavy weight. Another transducer type that was proposed for shear wave generation is based on the magnetostrictive effect [6,7,8,9]. These transducers also suffer from the disadvantage of a large mass due to the magnets that are needed for the generation of strong magnetic fields.

Several options are available for the generation of surface traction by piezoelectric elements [10]. For the generation of shear horizontal (SH) waves, the face and thickness shear modes are appropriate (see Figure 4 in Ref. [10]). A number of recent papers consider transducers based on these principles. For example, the thickness shear mode is studied in [11,12,13] and the face shear mode in [14,15,16,17,18]. The directivity characteristic of SHM transducers depends on the intended application. For surveying large areas with a small number of transducers, an omnidirectional characteristic is advantageous and therefore many papers aim for it [18,19]. On the other hand, a very high directivity can be beneficial for the investigation of a hot spot with good selectivity against noise that originates from other directions. In one recent publication, the authors propose a novel method for the construction of an SH0 wave transducer with high directivity that uses two adjoining face shear lead zirconate titanate (PZT) wafers [20].

Very flexible and lightweight piezoelectric transducers can be fabricated by dividing solid piezoelectric material into fibers and forming patches. These transducers were originally invented for adaptronic applications; however, they are now rather extensively studied as Lamb wave transducers [21,22], where three-dimensional (3D) Scanning Doppler Laser Vibrometry is used [23,24].

In the present contribution, we follow a recent work in which piezoelectric fiber patches (PFPs) are used to generate SH0 waves. A pure in-plane shear is generated by stacking two patches that operate in orthogonal directions and with the opposite sign [10]. This transducer type, called shear horizontal PFP (SHPFP), combines the high flexibility of PFPs with a pure in-plane shear operation. The operation’s basic principles were explained and confirmed by modeling and experimental verification [10,25]; however, there is much room for improvement.

This paper is organized as follows. In Section 2, we describe the application of PFPs to SH wave generation and define the performance criteria that we use in this work (Section 2.1). We propose two modified designs aiming to improve the SHPFP’s performance (Section 2.2). Both the modeling approach (Section 2.3) and the setup for experimental verification (Section 2.4) are described. In the results section (Section 3), we compare the performance of the modified versions with that of the original SHPFP with respect to the criteria SH0 mode purity and directivity. This is done by a numerical simulation of the transducers as well as by laser vibrometer measurements of the emitted signal amplitudes and wave fields. In Section 4, we discuss the results and ideas for the continuation of the research.

## 2. Materials and Methods

### 2.1. A Short Review of SH0 Wave Generation with the SHPFP

#### 2.1.1. Piezoelectric Fiber Patches (PFPs) for Guided Wave Generation

PFPs were originally developed for adaptronic applications; that is, sensing the deformation of a structure and actively deforming it [26]. The deformations are performed in a quasi-static way, meaning that the frequencies are so low that no elastic waves are excited.

**Figure 1:**
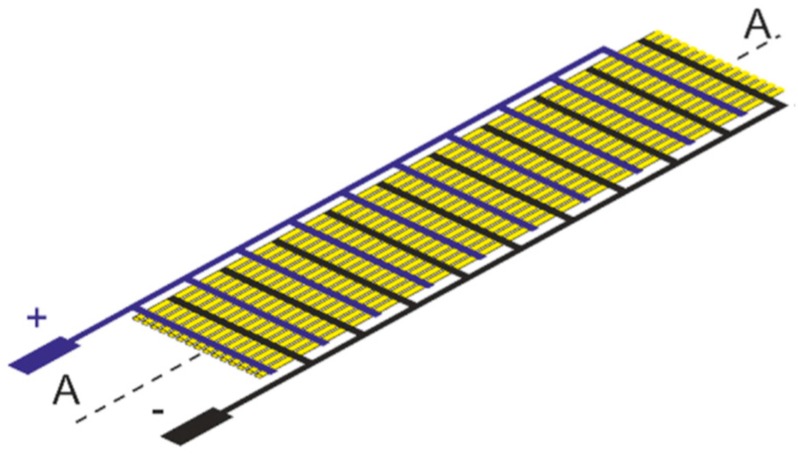
The scheme of a piezoelectric fiber patch (reprinted from Ref. [10] with permission from Elsevier). The finger-like electrodes are used for both the poling of the fibers and the generation of the electric fields in active operation.

**Figure 2:**
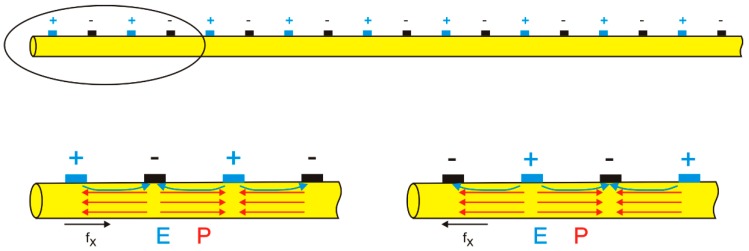
A cross-section of the piezoelectric fiber patch (PFP) shown in Figure 1 along the line A–A (a corrected version of Figure 4 in [10]). The upper row shows the fiber with electrodes; the lower row is a magnification of the left fiber’s end. The application of electric voltage in the two orientations leads to electric fields (E) that are antiparallel (left) and parallel (right) to the polarization (P). Simply speaking, a resulting force ($f_{x}\sim \sigma_{xx,x}$) is generated where the product $\sigma_{xx}\sim \;P_{x}E_{x}$ is changing from an average value to zero; that is, at the end of the polarized region of the fiber [22].

A PFP consists of piezoelectric fibers that are sandwiched between layers of adhesive, electrodes, and a polyimide film. The electrodes are oriented perpendicular to the piezoelectric fibers’ orientation and are arranged in an interdigitated pattern (Figure 1). The assembly enables in-plane poling, which can utilize the d_33_ effect for actuation. The polarization in each piezoelectric fiber is along the fiber’s direction with an alternating orientation (Figure 2). The applied electric field also has an alternating orientation so that the product of the polarization and the field has a constant sign (either positive or negative) along the fiber’s entire length with only some variation in amplitude. Assuming, for a moment, clamped conditions (i.e., there is no strain on the PFP due, for example, to gluing it onto a stiff substrate), the generated mechanical stress oscillates about a mean value along the fiber. This means that, on average, the forces that act on the substrate cancel out along the fiber and remain significant only at the fiber’s end.

The fiber patch’s performance depends on the average vector product of the polarization and the electric field that the electrodes introduce. Studies exist on how to optimize the performance by varying the electrode finger’s distance with respect to the thickness of the individual fibers [27]. Since the PFP’s design is now state-of-the-art and PFPs are available commercially, we will not go into these details.

Several authors have used PFPs for guided wave generation and detection in SHM. The mode of an excited wave will depend on the size of the patches and the used excitation frequencies [21]. The case of piezoelectric waves is similar, with the only difference being that the surface traction introduced into the substrate does not apply to all edges of the active transducer area but only to the edges of the fiber’s end. By slightly changing the electrode’s design and exciting each electrode group separately with certain time delay, the excited wave modes can be selected [22].

#### 2.1.2. The Working Principle and Configuration of the SHPFP

**Figure 3:**
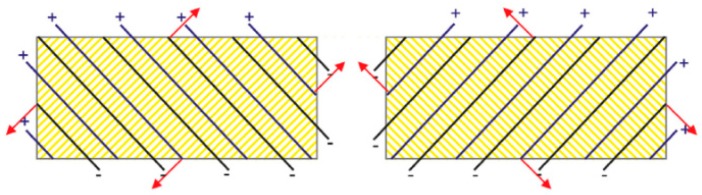
The two piezoelectric fiber patch (PFP) layers that overlie one another in a shear horizontal PFP (SHPFP) shown side-by-side. The red arrows indicate the resulting forces at the fiber ends of each individual layer (reprinted from [10] with permission from Elsevier).

**Figure 4:**
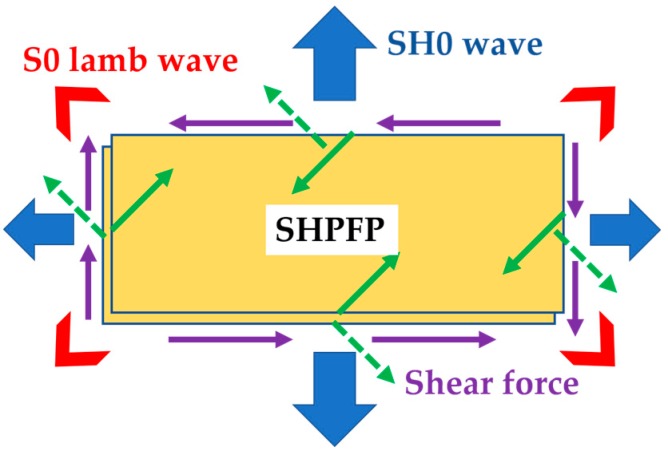
The configuration of the SHPFP. The yellow boxes show the active area of each PFP. The green arrows show the forces that each PFP generates. The underlying PFP generates the dotted green arrows and the overlying PFP generates the solid green arrows. At each edge, the dotted and the solid green arrow pairs are perpendicular to each other in-plane. The purple arrows designate the forces that result from the summation of the green arrows at each edge. The blue and red arrows represent excited wave modes, the target fundamental order of the shear horizontal mode (SH0) wave, and the unwanted S0 Lamb wave, respectively.

The SHPFP consists of two PFPs whose assembly design is different from that of a normal PFP (Figure 1). PFPs with a fiber and electrode orientation that is tilted ±45° relative to the PFP’s lengthwise orientation are used (Figure 3). One PFP overlies another PFP with an orthogonal fiber orientation and the two are excited simultaneously. We can use the two PFPs to generate shear forces parallel to their four edges by controlling the sign of the excitation signal at each PFP (Figure 4). This is how we can excite SH0 waves with PFPs and why the formed transducer is called SHPFP. The width of the SHPFP needs to be an odd multiple of the half wavelength of the desired SH0 wave, to enhance the constructive interference along the target direction. In addition, the length of the SHPFP is preferred to be multiple of the wavelength for the destructive interference along the unwanted direction.

#### 2.1.3. Optimization Criteria

The aim of the present study is to propose improved transducer designs. Thus, we need criteria to be able to measure the performance of the new designs versus that of the state-of-the-art. The criteria are defined with respect to the intended application, which is SHM with SH0 guided waves.

The first optimization criterion is purity, which is defined as the ratio of the amplitude of the target guided wave mode (SH0) to the next strongest amplitude of an unwanted (spurious) guided wave mode. By generating shear forces that are parallel to its edges, SHPFP excites SH0 waves through four different directions that are perpendicular to the edge orientations. However, when we consider the SHPFP’s corners, there are two shear forces that are perpendicular to each other. The summation of the two forces will generate not the target SH0 wave mode but the unwanted S0 Lamb wave mode. To obtain higher purity, the amplitude of the unwanted wave mode should be decreased.

The second criterion is directivity. Depending on the type of application, either an omnidirectional operation or a directional operation is advantageous. In this study, we optimized the transducer designs for a rather strong directivity. Among the four directions that the SHPFP excites SH0 waves through, there are two main directions from the length edges and two minor directions from the width edges. The two minor SH0 wave directions can increase the complexity of a signal’s interpretation in the main direction. Therefore, the SH0 waves in the minor directions should be suppressed to obtain better directivity.

### 2.2. Modified Designs

#### 2.2.1. Change in the Transducer’s Shape (Rounded Corner SHPFP)

The first proposed design aims to increase purity. A possible reason for the unwanted S0 Lamb waves could be right-angled corners. This idea comes from an analogy to other transducer types, where the edge of the aperture proves to be the source of additional wave modes. For normal non-destructive testing (NDT) bulk-wave transducers, it is well-known that transducers for longitudinal waves generate shear waves at the aperture’s edge [28] and transducers for shear waves generate longitudinal waves at the aperture’s edge. It is also known that appropriate aperture adopization reduces or avoids these edge waves [29]. Therefore, making the transition from an irradiated area to non-irradiated surroundings, less-sharp edges will reduce the number of unwanted contributions. The corners that terminate the radiating edges of our PFP correspond to the edge of the circular excitation area for a bulk-wave transducer.

**Figure 5:**
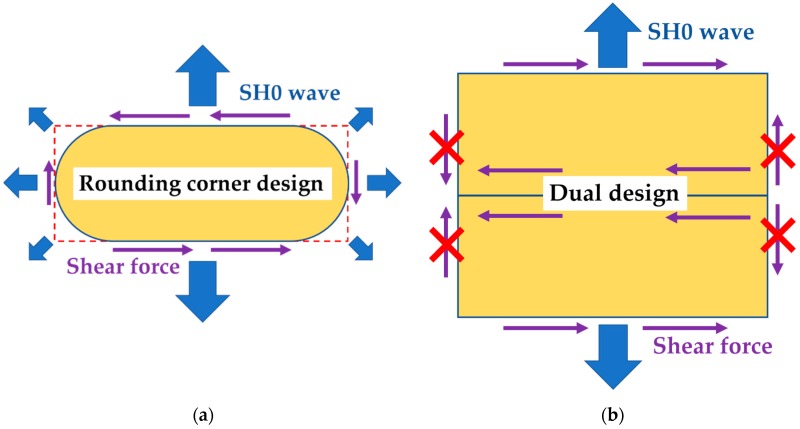
Two modified SHPFP designs. The purple arrows visualize the generated surface traction forces that act as shear forces, and the blue arrows indicate the excited wave modes. The left part shows (**a**) the rounded-corner design and the right part shows; (**b**) the dual SHPFP design. The red crosses in (**b**) indicate that the surface traction forces at the short edges act against each other and should (at least partially) cancel out. It should be noted that two PFPs overlie each other at each active area, even though the underlying and the overlying PFPs cannot be distinguished in the figure.

To determine whether the sharpness of the corners is crucial to the generation of S0 waves, a modified SHPFP design with rounded corners was investigated (Figure 5a). If the sharpness of the corners is essential for the generation of S0 waves, the “rounded corner” design would have a significantly increased purity ratio. For comparability, we chose the maximum dimensions in length and width of the original SHPFP. Because only the corners are removed, the fiber directions of each PFP is still maintained.

Of course, rounded-corner transducers cannot be obtained simply by cutting an original SHPFP into the new shape. The electrodes would be destroyed in such a process. The transducers have to be designed with a modified internal structure and produced accordingly.

#### 2.2.2. The Dual Transducer (Dual SHPFP)

The shear forces at the short edges of the original SHPFP not only decrease the directivity but also affect the purity. We obtain the second proposed transducer design by placing two original SHPFPs next to each other and operating them with opposite signs (see Figure 5b). This will increase the amplitude of the main SH0 wave and decrease the amplitude of the SH0 wave in the direction perpendicular to the main direction. This result is to be expected because the counteracting shear traction forces at the short edges of the PFP should (at least partially) cancel out in the far field of the transducer. Far away from the transducer and exactly on the symmetry line that is perpendicular to the short edges (the line where the two transducers in the dual design touch each other), the SH0 mode must be exactly zero due to the symmetry of the problem. The argument is independent of the frequency and size of the transducer; flipping the arrangement vertically leads to exactly the same excitation and, therefore, the same wavefield. This is possible for a vector in the vertical direction (such as the SH0 displacement on the horizontal axis) only when it is zero.

Hopefully, the counteracting traction forces will also decrease the number of unwanted waves that the corners emit. The expectation is that both the directivity and the purity of the dual SHPFP will be larger than those of the original SHPFP.

### 2.3. Finite Element Method (FEM) Simulation Setup

Numerical simulations were performed with the commercial FEM software ANSYS to validate the performance of the proposed designs. In the following subsections, we describe the validation of the PFP model’s implementation in ANSYS (Section 2.3.1) and the model of PFPs as guided wave transducers (Section 2.3.2).

#### 2.3.1. Homogenized PFP Model Verification

The first step in the simulations was to decide how to model a PFP. A PFP consists of piezoelectric fibers and two electrode layers with an interdigitated pattern. Due to this complexity, it is not only difficult to model its exact structure but an implementation that captured the structure’s details would lead to a computationally very inefficient calculation.

**Figure 6:**
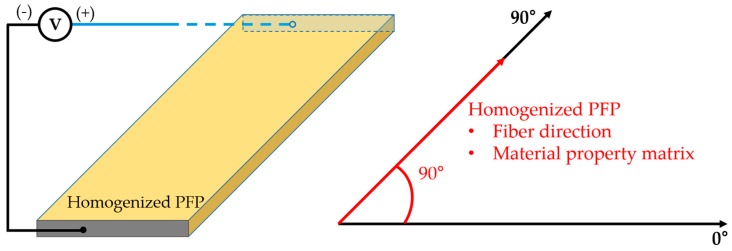
The schematic configuration of the homogenized PFP with its voltage boundary condition. The red arrow shows the direction of piezoelectric fibers in the PFP, and it also indicates the orientation of the material property matrix (piezoelectric, dielectric, and stiffness coefficients) of the homogenized PFP.

**Figure 7:**
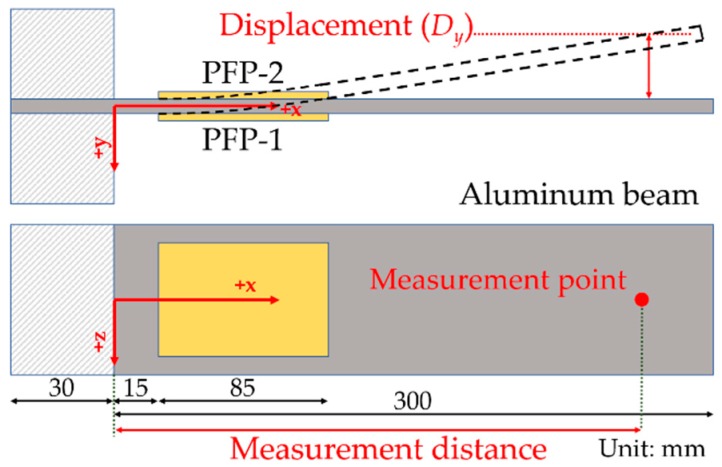
The homogenized PFP bending test’s configuration. Two PFPs (85 × 57 mm^2^ in size) were attached to the front and the back of an aluminum beam. The PFP1 was driven by a voltage to be extended, while the PFP2 remained as a closed-circuit. In the experiment, 30 mm of the 330-mm-long aluminum beam was fixed by a clamp [31]. In the simulation, the aluminum beam was modelled with a length of 300 mm and with fixed boundary conditions at the left end.

Fortunately, an effective approach to modelling a PFP’s behavior is available [30,31]. A PFP is considered to be a homogenized but anisotropic material with effective piezoelectric properties. Here, anisotropy is present in the piezoelectric coupling between the electric and mechanical fields, as in poled piezoelectric discs, and there is strong anisotropy in the mechanical properties (stiffness anisotropy) and in the dielectric properties. The configuration of the homogenized PFP is shown in Figure 6. The red arrow is parallel to the piezoelectric fibers’ direction, and also indicates the standard orientation of the material’s tensor components (which we also call the material property matrix). This means that the piezoelectric fibers are aligned along the PFP’s lengthwise direction. In this homogenized model of the PFP, both electrodes are assumed to be on the short sides, which leads to a constant electric field over the whole PFP. This approach has already been verified by a numerical simulation of performed experiments. What remains to be shown is that our implementation of the approach in ANSYS is correct. We did that by reproducing the experimental results of [31] using ANSYS. Figure 7 provides the details of the experimental situation. Two normal PFPs (M8557P1, Smart Materials GmbH) were bonded to the front and back surface of an aluminum beam (330 × 17 × 1.97 mm^3^). To make the displacement, the PFP1 was driven by a voltage, while a closed-circuit boundary condition was imposed on PFP2. The material properties that were used in the studies [30,31] and in our numerical simulation are shown in Table 1.

**Figure 8:**
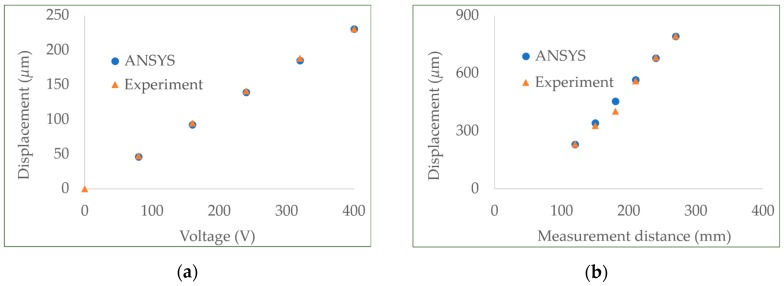
Comparison of the results from the homogenized PFP bending test. (**a**) The measurement point location was fixed, and the applied voltage was changed; (**b**) The applied voltage was fixed and the measurement distance was changed. The blue dots show the simulation results and the orange triangles show the corresponding experimental results [31].

In the first test, we followed the displacement of the beam at a fixed measurement point as the voltage applied to the PFPs increased (Figure 8a). In the second test, we fixed the applied voltage and changed the measurement point’s location (Figure 8b). The perfect agreement of the results from both models with the published experimental values proves the reliability of the homogenized PFP approach in our implementation.

#### 2.3.2. Modeling Details

**Figure 9:**
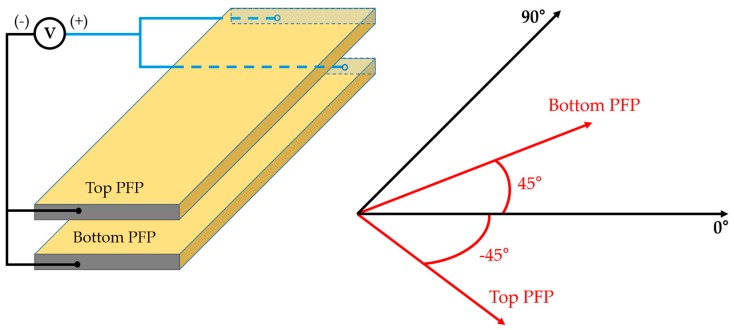
The schematic configuration of the homogenized original SHPFP with its voltage boundary condition. The red arrows indicate the direction of piezoelectric fibers and the rotated material property matrix of each homogenized PFP.

The same homogenized PFP approach as in the validation example was used to model the new and the reference SHPFP designs (Figure 9). The PFP was placed in the model onto a steel plate measuring 500 × 500 × 2 mm^3^. Its Young’s modulus, density, and Poisson’s ratio were 200 GPa, 7850 kg/m^3^, and 0.30, respectively. The original SHPFP was made of two homogenized PFPs with different tilted fiber directions (+45° and −45°). The material property matrix was also rotated +45° and −45° for the bottom and top PFPs, respectively, compared to that of the standard PFP shown in Figure 6. A perfect bond between both PFP layers and between the bottom layer and the substrate was assumed. This means that all displacement components $u_{i}$ and normal stress components $\sigma_{zi}\;\left(i=1,\;2,\;3\right)$ are continuous across the interface.

**Figure 10:**
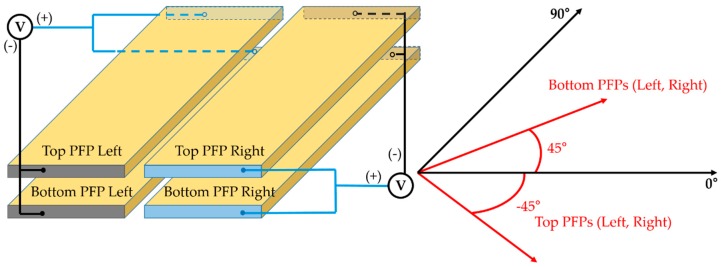
The schematic configuration of the homogenized dual SHPFP with its voltage boundary condition. The two voltage sources are exactly the same. The red arrows indicate the direction of the piezoelectric fibers and the rotated material property matrix of each homogenized PFP.

The size of each PFP was 40 × 10 × 0.3 mm^3^. The rounded corner design was modeled with the same length (40 mm) and width (10 mm) value of the original SHPFP design. It should be noted that the rounded corner design has a smaller active area compared to the original design. The dual SHPFP design was made of two SHPFPs. Each homogenized PFP had its own fiber direction and a rotated material property matrix (Figure 10). Again, perfect bonding interface conditions between the layers and between the bottom layer and the substrate were assumed. Because each SHPFP has a size of 40 × 10 × 0.6 mm^3^, the dimensions of the dual design were 40 × 20 × 0.6 mm^3^.

**Figure 11:**
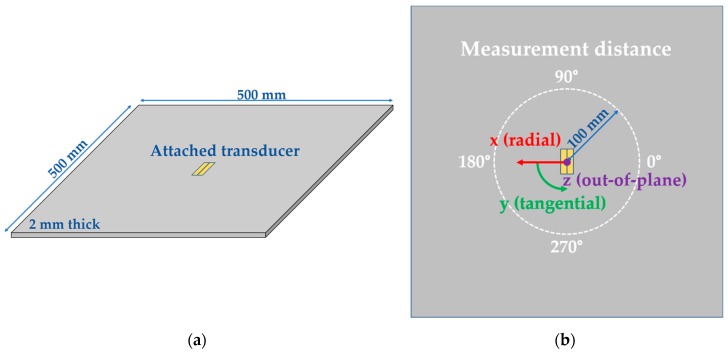
The modeling configuration. (**a**) General view; (**b**) top view. The size of the steel plate was 500 × 500 × 2 mm^3^, and the transducers were always located at the center. The red, green, and purple arrows indicate the radial (x), tangential (y), and out-of-plane (z) axes of the cylindrical coordinate system. The symbols (x,y,z) for the cylindrical coordinates were chosen to be in accordance with the output of ANSYS (compare Figure 14). The emitted waves were measured at a distance of 100 mm from the center.

All transducers were located at the center of the steel plate and the emitted wave was measured in different directions at points on a circle at a distance of 100 mm (the white dotted line in Figure 11b). To ensure that the simulation results were reliable, the mesh size was set to be smaller than *λ*⁄10 and the time step (∆*t*) was set to be smaller than 1/(10*f_c_*). Thereby, *f_c_* is the center frequency of a three-cycle, Hanning-windowed sinusoidal tone burst, which was used as the exciting signal, and *λ* is the wavelength of a horizontal shear wave in the plate at *f_c_*. The voltage of the applied signal was chosen to have an electric field of 200 V/mm through each PFP and its center frequency (*f_c_*) varied from 50 kHz to 200 kHz.

**Figure 12:**
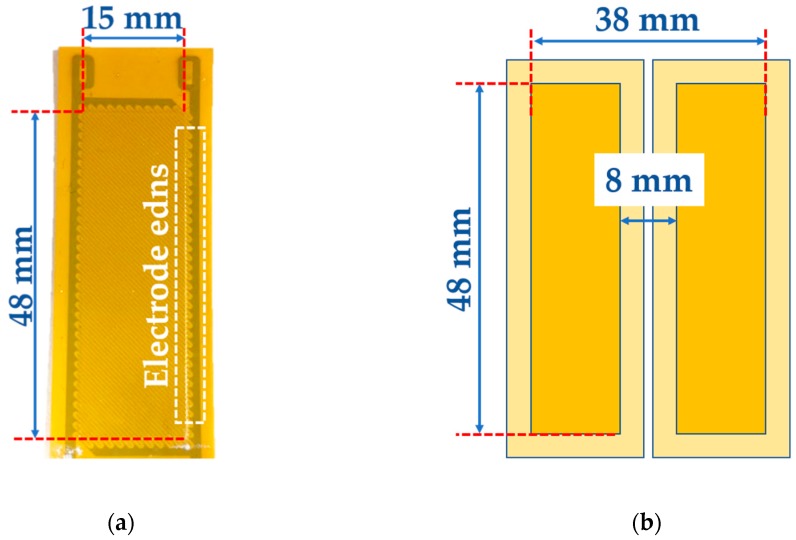
Realization of the dual SHPFP design. (**a**) An actual photo of the commercial PFP (M4815F1, Smart Materials GmbH) that was used to build the dual design. The size of the active area is 48 × 15 mm^2^ and the PFP has electrode ends at both of its long edges; (**b**) The configuration of the realized dual SHPFP design. Because of the electrode ends, there is an 8-mm gap between the SHPFP on the left side and the SHPFP on the right side.

An additional dual SHPFP simulation was performed in order to conduct a direct comparison between the simulation and experimental results. Due to restrictions on the available PFPs, the ideal geometry could not be realized in the experimental setup. The size of each PFP was changed to 48 × 15 × 0.3 mm^3^ and there was an 8-mm gap between the two SHPFPs that formed a dual SHPFP (Figure 12). Details are provided in Section 2.4.

### 2.4. Experimental Setup

Experiments were conducted to validate the simulation results. A Laser Doppler Vibrometer from Polytec GmbH was used to record the full vector of the surface particle’s velocity. To enhance the amount of light that was backscattered into the vibrometer’s heads, a retroreflective foil with a size of 574 × 560 mm^2^ was attached to the plate. Each measured signal was averaged over 128 excitations to improve the signal-to-noise ratio.

We used an in-house-developed Multichannel Acoustic Measurement System (MAS-2) to generate the excitation voltage. It is designed for both passive acoustic emission measurements and active guided wave measurements and has four channels. We used one channel of the active part. The arbitrary waveform generator uses 500 k samples and has a digital-to-analog converter with 14-bit resolution. The output frequency is 18.75 MHz and the maximal output voltage is 160 Vpp at a load of 10 nF. The MAS-2 triggers the Laser Doppler vibrometer system, which delivers a three-channel dataset for the time signal of the three displacement velocity components at each measurement point.

Exact realizations of the proposed designs would be costly. In particular, changing the shape of a PFP to build the rounded-corner SHPFP would involve a lot of effort. Considering the insignificant improvement in performance that was obtained with this design in the simulations (see Section 3), the benefit is marginal. Therefore, experiments with the rounded-corner design were not conducted in this study.

Two PFPs are needed to form the original SHPFP and two SHPFPs are needed to build the dual SHPFP design. Therefore, the dual SHPFP design was made out of four commercially available PFPs (M4815F1, Smart Material GmbH). Honey was used as a coupling material to keep the PFPs detachable and magnets were placed on top of the dual SHPFP to enhance the bonding quality. It should be noted that this workup approach is less perfect as compared to having one complete housing package for the dual SHPFP. Specifically, in this workup, the upper layer of the dual SHPFP should transfer its forces through the coupling layer and the lower layer. This separation of the upper layers and the lower layers can affect the obtained results. Another limitation has its source in the separate housing of the PFP. Each actual PFP has electrode ends at its long edges (Figure 12a). Therefore, the active area of the left SHPFP cannot be perfectly bonded to the active area of the right SHPFP, and there is an intrinsic gap between the active areas of the two SHPFPs (Figure 12b). The size of the dual SHPFP will not be 48 × 30 × 0.6 mm^3^, but 48 × (30 + 8) × 0.6 mm^3^, because of the 8-mm gap between the two SHPFPs (Figure 12b). This problem can be solved by putting the electrode ends at the short edges (see Figure 7 in Ref. [10]), however this will not be discussed in this study.

The dual SHPFP workup was mounted on a 2 mm steel plate with a size of 1000 × 1000 mm^2^. The PFPs were excited by a three-cycle, Hanning-windowed sinusoidal tone burst, as was done in the simulations. We chose a 200 V peak-to-peak voltage value to maintain the PFP’s electric field at 200 V/mm. Considering a shear wave velocity in steel of 3250 m/s, a center frequency of 80 kHz was chosen to match the wavelength *λ* of the SH0 wave to the width of the dual SHPFP.

## 3. Results

### 3.1. Results from the Simulation of the Modified Designs

#### 3.1.1. Rounded-Corner SHPFP

**Figure 13:**
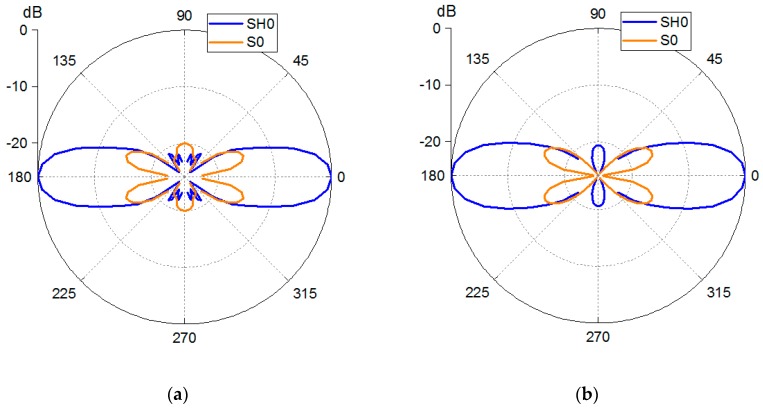
A normalized polar diagram of the wave amplitude (particle velocity) of (**a**) the rounded-corner SHPFP and (**b**) the SHPFP. The blue lines show the SH0 wave amplitude, while the orange lines show the unwanted S0 Lamb wave amplitude.

Figure 13 shows a normalized time domain wave amplitude of the simulated velocity components of the original SHPFP (Figure 13a) and the rounded corner SHPFP (Figure 13b) in a cylindrical coordinate system at a center frequency of 160 kHz. Since the particle movement of the SH0 wave is purely in-plane and perpendicular to the direction of propagation, the tangential components in the cylindrical coordinate system will represent the SH0 wave alone to a good approximation. In a similar manner, the S0 wave is mainly (but in this case not exclusively) in-plane in the propagation direction. Therefore, we label the tangential and the radial components of the displacement velocity in Figure 13 and all the following polar diagrams by SH0 and S0 amplitudes, respectively.

It is quite clear from the images that both the SHPFP and the rounded-corner design mainly excite the SH0 waves into two opposite directions (0° and 180°). The second-largest amplitude wave mode is the S0 Lamb wave (the radial component).

By changing the design from sharp corners to rounded corners, the maximum amplitude of the SH0 wave increased by 8.83%, and the maximum amplitude of the S0 Lamb wave increased by 3.13%. Therefore, the purity ratio (the SH0⁄S0 amplitude ratio) increased by only 5.53% (from 5.737 to 6.054). This is an improvement, but it is barely visible in Figure 13. Considering the effort that is required to change the design and to produce the more complex transducer, the obtained benefit is marginal.

#### 3.1.2. Dual SHPFP

**Figure 14:**
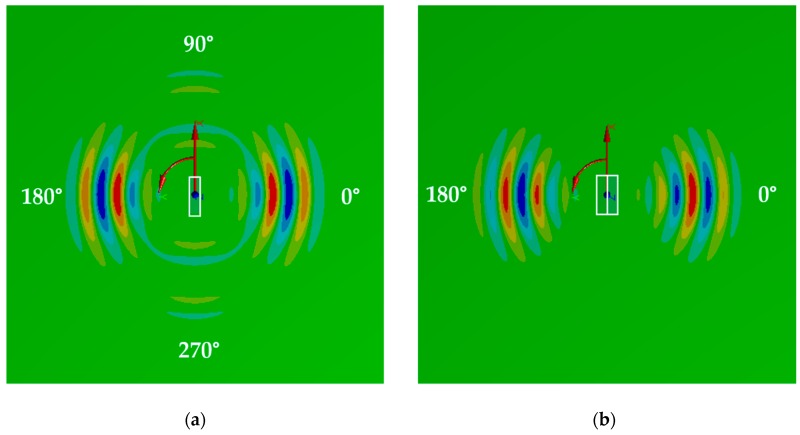
The tangential component of the simulated wave field at a time of 50 µs for (**a**) the original SHPFP and (**b**) the dual SHPFP. The excitation has a center frequency of 80 kHz. For the complete videos of the wave propagation, see the section Supplementary Materials at the end of the paper.

**Figure 15:**
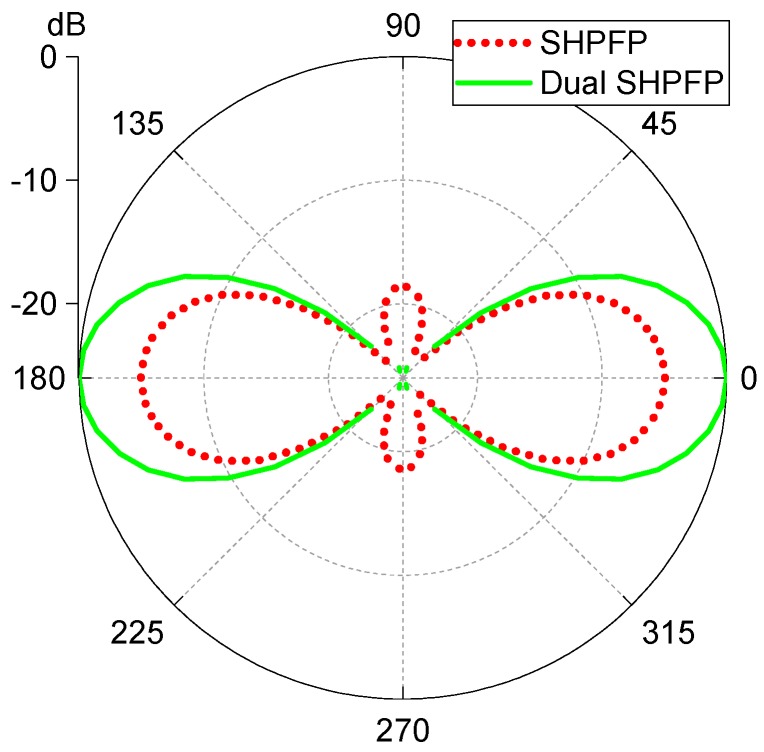
A comparison of the SH0 wave directivity of the SHPFP (the red dotted line) versus the dual SHPFP (the green solid line) at a center frequency of 80 kHz. The values were normalized to the maximum value of the dual SHPFP transducer.

**Figure 16:**
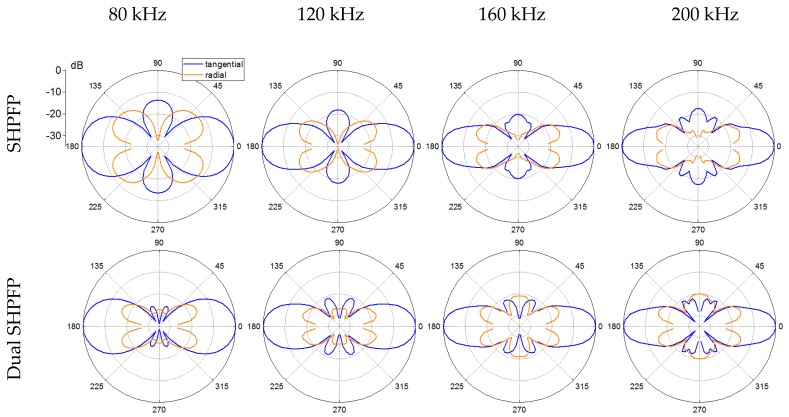
Polar plots of the maximum values of the surface velocity components. The plots of the original SHPFP and the dual SHPFP are compared for different center frequencies. The blue lines show the SH0 wave’s amplitude and the orange lines show the unwanted S0 Lamb wave’s amplitude. The values in each polar diagram were normalized to the maximum value of each graph. The scale is in dB and all values below −35 dB are cut off.

First, we consider the directivity of the dual SHPFP. Figure 14 shows a snapshot of the tangential wave field component (SH0 wave) of the original SHPFP (Figure 14a) and the dual design (Figure 14b). Both simulations were performed with an 80 kHz center frequency excitation. It can be seen from Figure 14a that the SH0 wave is excited not only through the main directions (0° and 180°), but also through the unwanted directions (90° and 270°). In comparison, only the main wave packets are visible in Figure 14b. Other wave packets that are perpendicular to that might be present, but they are too weak to be visible in the image. A polar diagram of the tangential components of the original and the dual design were prepared for a quantitative comparison (Figure 15). The values were normalized to the maximum of the dual SHPFP. As shown in the diagram, the dual SHPFP generates SH0 waves along the main directions, while the original SHPFP generates SH0 waves along both the main and the minor directions. Of course, the SH0 directivity of both transducer variants depends on the center frequency. While this dependence is not the focus of this paper, we will discuss it briefly with help of Figure 16. The values were normalized to the maximum of each individual plot, which is, in all cases, the 0° (or 180°) direction of the tangential displacement velocity component (SH0). Concentrating on the blue lines of the SHPFP (the upper row), the best ratio between the main lobe at 0° and the unwanted 90° lobe (20 dB) is at *f* = 160 kHz. This is not unexpected, because, at that frequency, the patch’s width matches half the shear wavelength in the plate, so there is a wavelength-matched excitation in the 0° direction. There is no matching in the 90° direction at any frequency. For the dual SHPFP, the frequency dependence is more complex and will not be discussed further. Considering the lobe around 90° of the dual SHPFP’s tangential component in the logarithmic scale, the reduction to zero just on the 90° axis is remarkable. This is obviously due to the symmetry of the excitation, as we have already discussed in Section 2.2.2. This enforced zero is connected with a reduced size of the entire 90° lobe in the dual SHPFP.

The next criterion is purity. As discussed in Section 2, the shear forces on the width side of the SHPFP and the corners can be considered to be one of the main sources of S0 Lamb wave generation. Since the surface traction forces along the width sides of the dual design work against each other and (at least partially) cancel each other out, the amplitude of the generated S0 Lamb wave is expected to be smaller. To confirm this expectation, we compared the SH0 and S0 Lamb waves for both transducer types. Figure 16 shows the SH0 wave (blue) and the S0 Lamb wave (orange) at different center frequencies. It is clear that both the directivity and the purity have been improved with the proposed dual design. To quantitatively evaluate the improvement in purity, the purity ratio was calculated for each case. The maximum purity ratio of the original SHPFP at a center frequency of 120 kHz was found to be 6.010. The dual design shows a maximum purity ratio of 10.707 at a center frequency of 100 kHz (Table 2). It should be noted that, through a wide range of center frequencies, the purity ratios of the dual SHPFP are always higher, which indicates that the dual design can generate an almost pure SH0 wave through different driving frequencies.

#### 3.1.3. Dual SHPFP with an 8-mm Gap

An additional dual SHPFP simulation was done in order to conduct a direct comparison between the simulation results and the experimental results. All other conditions in the simulation were identical to those in the former dual SHPFP simulations. The only differences were that the size of each PFP was 48 × 15 × 0.3 mm^2^, rather than 40 × 10 × 0.3 mm^3^, and there was an 8-mm gap between the two SHPFPs in the dual SHPFP (Figure 9).

**Figure 17:**
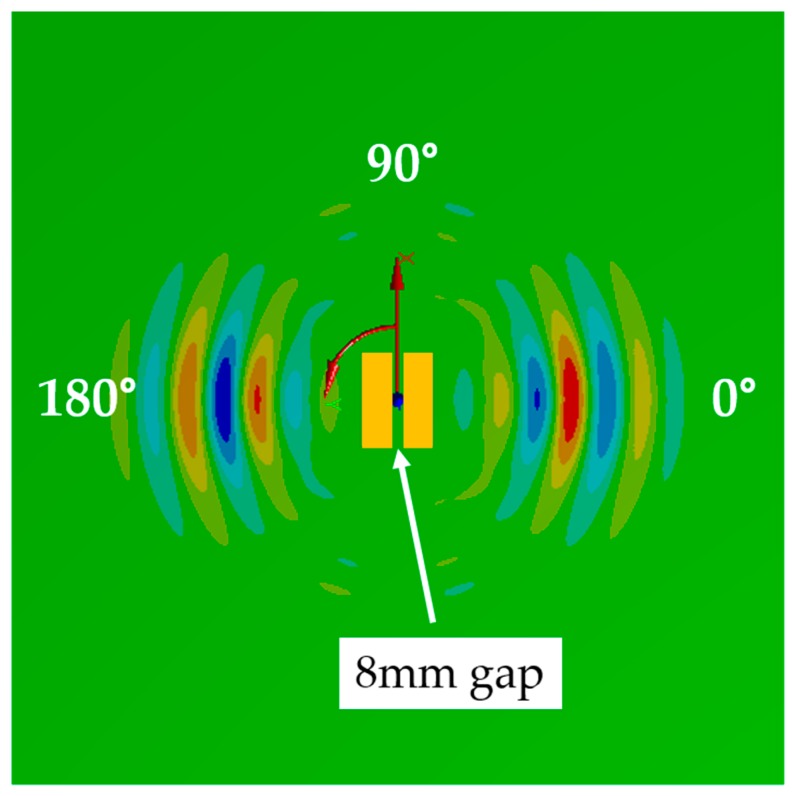
Result of the simulation of the dual SHPFP with an 8-mm gap. Snapshot of the tangential wave field component at 50 µs after excitation with $f_{c}=80\;\mathrm{kHz}$.

Even though there was an 8-mm gap, the dual SHPFP was able to generate clear SH0 waves through main directions (Figure 17). Because it is not a perfect dual SHPFP configuration, small visible wave packets can be observed near the minor directions. However, the relative amplitudes of the small wave packets are insignificant. The calculated purity ratio was 3.81; this value will be compared with the experimental result in the next section.

### 3.2. Experimental Results

**Figure 18:**
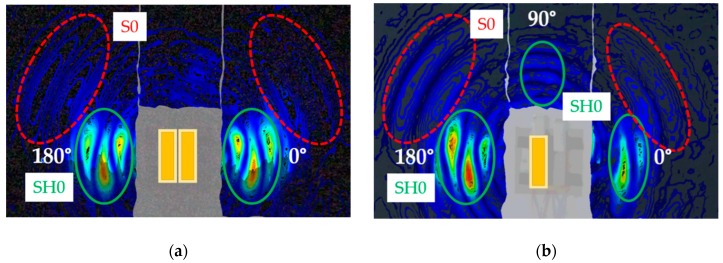
A comparison of measured wave field snapshots between (**a**) the dual SHPFP and (**b**) the original SHPFP at 50.24 µs after excitation. The wave packets in the solid green circles are the SH0 waves, and the ones in the dotted red circles are the unwanted S0 Lamb wave. For the complete videos of the wave propagation, see the section Supplementary Materials at the end of the paper.

Figure 18 shows a snapshot of the wave fields that were generated by the dual SHPFP (Figure 18a) and the original SHPFP (Figure 18b). For the complete videos of the measured wave propagation, see the Supplementary Materials cited at the end of the paper. The four PFPs were attached to the steel plate to form the dual SHPFP and the scanning area was fixed. To generate waves with the dual design, we applied the voltage signal to all four PFPs. When we operated only the two PFPs on the left-hand side (the other two on the right-hand side were still attached but not under excitation), they functioned as the original SHPFP. As expected, the original SHPFP generates SH0 wave packets in the main direction and less-intense wave packets in the minor direction (90°), while the dual SHPFP generates SH0 wave packets primarily along the main direction. The original SHPFP clearly generates the unwanted S0 Lamb wave. Even though the S0 Lamb wave packet is still visible in the dual SHPFP graph, the relative amplitude is reduced. It should be mentioned that an unexpected phenomenon can be observed in Figure 18. The figure shows that the maximum amplitudes of the SH0 wave packets along the 180° direction do not lie on a center line. This was not present in the modeling result. This asymmetry disappears at a later time in the wave’s propagation. Even though the reason for the wavering maxima is not clear, we attribute it to the imperfection in the experiment. One possible explanation is related to what was mentioned in Section 2.4 about ‘transferring the surface traction of the upper-layer PFP through to the bottom-layer PFP’. The forces that the upper layer generates should travel through the lower layer to reach the substrate. We believe that, because of this through-thickness operation, the upper layer’s traction is delayed in a timely manner compared to that of the lower layer’s traction. Therefore, the maxima do not lie on a single center line but rather waver over the line.

**Figure 19:**
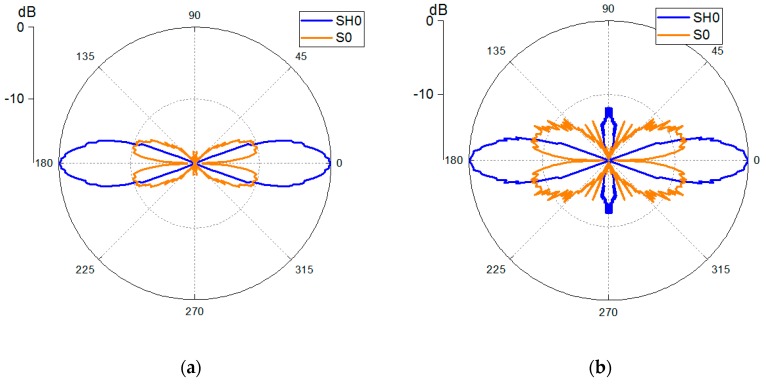
Polar diagrams showing the SH0 amplitude (blue lines) together with the S0 Lamb wave amplitude (orange lines) of (**a**) the dual SHPFP and (**b**) the original SHPFP.

A more quantitative comparison of both transducers is possible with the polar diagrams in Figure 19. Figure 19a,b shows the dual SHPFP results and the original SHPFP results, respectively. The blue lines and the orange lines represent the velocity of the tangential component (SH0) and the radial component (S0), respectively. It is clear that the amplitude of the S0 Lamb wave is much higher in the original SHPFP. The purity ratio was calculated for each transducer. The dual SHPFP shows a purity ratio of 3.19, while the original SHPFP shows a purity ratio of only 2.48. It should be noted that the purity ratio result from the additional dual SHPFP simulation (with an 8-mm gap and an 80 kHz center frequency signal) was 3.81. Considering that coupling in the experiment could not be perfect, the results from the experiment (3.19) and from the simulation (3.81) are quite comparable.

**Figure 20:**
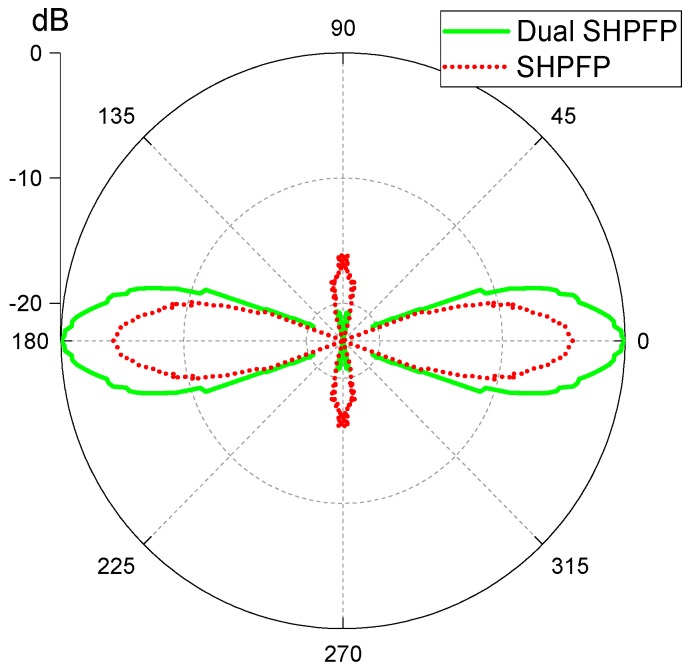
A polar diagram for the direct comparison of the original SHPFP and the dual SHPFP as a SH0 wave transducer. The solid green and the dotted red lines show the SH0 amplitude of the dual SHPFP and the original SHPFP, respectively. The values are scaled to the maximum of the dual SHPFP.

An even clearer comparison between the original SHPFP and the dual SHPFP as an SH0 wave transducer is shown in Figure 20. The solid green line shows the tangential velocity that the Dual SHPFP generates, and the red dotted line shows the tangential velocity that the original SHPFP generates. The maximum velocity increment was 160%.

## 4. Discussion and Conclusions

We started this study by explaining recent work on the generation of SH0 waves with PFPs, the so-called SHPFP. Modified designs that aimed for improved performance were proposed. Two optimization criteria were used: purity and directivity.

The validity of the modified designs was tested with simulations. The rounded-corner SHPFP showed a slight change in purity that could not be interpreted as a significant improvement. Thus, the idea that the sharp corners of the SHPFP are responsible for the S0 contributions was essentially disproved. It makes little sense to put considerable effort into changing the design and production of the patch when the idea has already been disproved in the model. The dual SHPFP showed a significant improvement in both purity and directivity. The validity of the dual SHPFP concept was tested over a wide frequency range (from 80 kHz to 200 kHz).

The validity of the dual SHPFP as an SH0 wave transducer was tested in simulations, and an experimental realization with commercial PFPs was conducted. Because of the electrode ends at the edge of each PFP’s length side, an 8-mm gap between the active areas of the two SHPFPs was inevitable. To perform a direct comparison with the experimental result, additional simulations of the dual SHPFP with an 8-mm gap and the exact dimensions of the active areas were done. Despite the 8-mm gap, the realized dual SHPFP showed good performance compared to the original SHPFP.

Even though the simulations and the experiments were done on a metal plate, one can also expect a better performance on non-magnetic as well as composite plates using the investigated dual SHPFP. This is clearly an advantage compared to common ways to excite shear waves (EMATs), because the SHPFP is not limited to magnetic materials.

As mentioned in the introduction, there are also lightweight shear wave transducers that are based on face shear PZT wafers. One advantage of SHPFP over PZT wafers is the flexibility of the fiber patches, which allow for its application to curved surfaces. To date, no comparison of both concepts with respect to the efficiency of wave generation has been done. Such a study would be very interesting, particularly if the transducer proposed by Miao [20] were to be compared with the dual SHPFP proposed in this work. Unfortunately, such an investigation goes beyond the scope of this paper and must be left to later work.

We investigated the proposed transducers in their capacity as transmitters, that is, in their capacity to produce a field of guided waves in a structure after excitation by an electrical signal. Of course, in any NDT and SHM application, the transducer is operated in both ways: as a transmitter and as a receiver. So, in principle, we should also study the transducers in their capacity as sensors. Fortunately, this is not necessary to perform separately. The reciprocity theorem [32] states that, in short, the transmission transfer function of a piezoelectric transducer is (up to a factor) equal to its receiving transfer function. To be very precise, the theorem has to be translated to each specific situation, e.g., by specifying the electrical termination and defining what is to be measured on both the electrical side and the mechanical side of the transducer. This exact analysis is seldom done in the literature. We share the position with many scholars that the reciprocity is fulfilled to a very good approximation under normal operating conditions and assume that this is true also for our specific situation.

Further work will focus on the SHPFP and extend the dual SHPFP such that the number of acting elements exceeds 2. This can be done by individual excitation of several electrode finger groups. Similar approaches have already been demonstrated for the Lamb wave PFP [33,34], and will be transferred to the SHPFP.

## Figures and Tables

**Table 1:** The effective material properties of the homogenized M8557P1 PFP [31]: Young’s modulus (*E*), shear modulus (*G*), Poisson’s ratio (γ), piezoelectric coupling coefficients ($d_{ij}$), and relative permittivity at constant strain ($\varepsilon_{ij}^{s}$).

Properties	Value
$E_{33}$ (GPa)	29.4
$E_{11}$ (GPa)	15.2
$G_{31}$ (GPa)	6.06
$\gamma_{31}$	0.312
$\gamma_{13}$	0.16
$d_{33}$ (pm/V)	467
$d_{32}\;$ (pm/V)	−210
$d_{31}$ (pm/V)	−210
$\varepsilon_{11}^{s}$	712
$\varepsilon_{22}^{s}$	1.7
$\varepsilon_{33}^{s}$	737

**Table 2:** Comparison of the purity ratio at different center frequencies.

$\mathrm{Center}\;\mathrm{Frequency}\;f_{c}/kHz$	SHPFP Purity Ratio	Dual SHPFP Purity Ratio	Increment
80	3.977	6.493	160%
100	5.304	10.707	202%
120	6.010	7.427	124%
140	5.372	7.053	131%
160	5.488	6.081	111%
180	5.452	5.814	107%
200	5.477	5.595	102%

## Supplementary Materials

The following files are available online at: https://zenodo.org/record/2654515#.XP8scmMuCr. All simulations and measurements are performed at a center frequency of 80 kHz. DualSH-PFP_measurement.avi: velocity magnitude generated by the Dual SHPFP and measured by Laser Doppler vibrometry. SH-PFP_measurement.avi: velocity magnitude generated by the original SHPFP and measured by Laser Doppler vibrometry. DualSHPFP_simulation.avi: circumferential component of the velocity generated by the Dual SHPFP and determined by simulation. SHPFP_simulation.avi: circumferential component of the velocity generated by the original SHPFP and determined by simulation
